# Role of MicroRNA-30c in cancer progression

**DOI:** 10.7150/jca.38449

**Published:** 2020-02-14

**Authors:** Wenyan Han, Hongwei Cui, Junqing Liang, Xiulan Su

**Affiliations:** 1 Laboratory of the Second Affiliated Hospital of Inner Mongolia Medical University.No.1 Yingfang Road, Huimin District, Hohhot, Inner Mongolia, China.; 2 Clinical Medical Research Center of the Affiliated Hospital/Inner Mongolia Key Laboratory of Medical Cellular Biology, Inner Mongolia Medical University, Hohhot, 010050, Inner Mongolia, P.R. China; 3 Department of Breast Oncology, Inner Mongolia Autonomous Region Cancer Hospital, Hohhot, 010000, Inner Mongolia, P.R. China

**Keywords:** microRNAs, microRNA-30c, cancer progression, cancer treatment, cancer prognosis

## Abstract

MicroRNAs (miRNAs or miRs) is a non-coding small RNA of a type of 18~24 nucleotide-regulated gene that has been discovered in recent years. It mainly degrades the target gene mRNA or inhibits its translation process through the complete or incomplete bindings with 3'UTR of target genes, followed by the regulation of individual development, apoptosis, proliferation, differentiation and other life activities through the post-transcriptional regulation. Among many miRNAs, the microRNA family, miR-30, plays diverse roles in these key process of neoplastic transformation, metastasis, and clinical outcomes in different cancer progression. As key member of miR-30, miR-30c is regulated by oncogenic transcription factors and cancer progression related genes. Recently, numerous studies have demonstrated that the aberrant expression of miR-30c was significantly associated with the majority of human cancer progression. In this review, the diverse roles of miR-30c in different cancer progression such as the cellular and molecular mechanisms, the potential applications in clinics were summarized to speculate the benefits of miR-30c over-expression in cancer treatment and prognosis.

## Introduction

MicroRNAs (miRNAs or miRs)are endogenous, non-coding single-stranded small-molecule RNAs of a class of 18 to 24 nucleotide-regulated genes. The specific bindings between miRNAs and the 3' untranslated region (3'UTR) of the target mRNA lead to the degradation or translational inhibition of the target mRNA, which results in post-transcriptional gene silencing. The specific associations between miRNAs and target mRNA depends on the complementarities between miRNAs and the target mRNAs. The degradation of target mRNA occurs when complementarities is complete or nearly complete, which is more common in plant miRNAs, while most mammalian miRNAs inhibit the post-transcriptional translation of target mRNA by the formation of incomplete complementary sequence 1,2.

In human beings, the majority of miRNAs are located in introns or non-coding mRNA transcripts of protein-coding genes, while the remaining miRNAs are distant from the transcripts in the genome, such as the exons of non-coding mRNA genes, or 3' UTR of mRNA genes, or other miRNAs genes^3^. In recent years, more and more studies have proved that miRNAs play important roles in a variety of cancer progression, such as respiratory cancers^4^, digestive system cancers^5^,^6^, neurological system cancers^7^, genitourinary cancers^8^ and vascular cancers^9^. miRNAs regulate the proliferation, differentiation, invasion, apoptosis and metastasis process of cancer cells in the form of oncogenes or tumor suppressor genes^10^,^11^.It is worth noting that miRNAs and their target mRNA play synergistic network roles in cancer progression, and the analyses of miRNA expressions have identified potential markers for the diagnosis, prognosis and treatment of cancer patients^12^,^13^.In these respects, more and more studies have confirmed that microRNA-30 family members maintain low expression levels in different cancer tissues and play a tumor suppressor role in cancer progression, metastasis and drug resistance^14^,^15^.In this review, the role of microRNA-30c (miR-30c) in cancer progression was summarized to address the consequences associated with reduced expression levels of miR-30c and speculate on the benefits of miR-30c over-expression in cancer therapy.

## The biogenesis of microRNA

microRNAs (miRNAs or miRs) are endogenous non-coding single stranded small RNA of 20-24 nucleotides in *eukaryotes*. In 1993, Lee *et al* firstly reported a 22-nucleotides-long non-coding small RNA, LIN-4, which regulates the development of *Caenorhabditiselegans*^16^. Since then, a variety of microRNAs have been found. MicroRNAs play important roles in signal transduction, cell differentiation, proliferation, apoptosis, blood vessel formation and development, as well as inflammation and tumorigenesis *in vivo*. In *eukaryotes*, microRNAs are located in the non-coding region of genome, meanwhile, majority of microRNAs of human beings are located within introns or non-coding mRNA regions in genome with a portion of microRNAs located far from the transcribing regions^17^.

**Figure 1:**
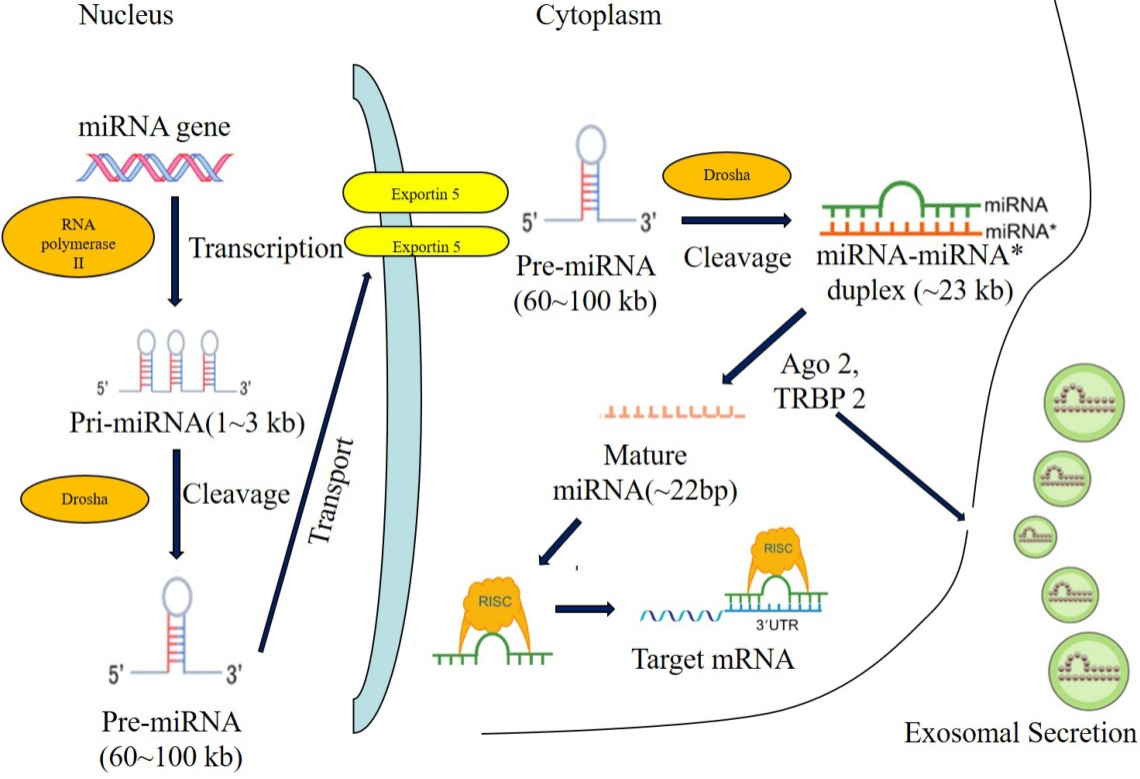
** The biogenesis of miRNA.** Note: Most miRNAs are transcribed by RNA polymerase II (RNA pol II) in the form of primary-miRNA (pri-miRNA), and then processed in the nucleus by Drosha andcleavaged to precursor miRNA (pre-miRNA). The pre-miRNA is further exported to the cytoplasm by exportin5 and further cleavaged by a complex containing Ago 2 and TRBP 2. The functional strand of mature miRNA is incorporated into the RNA-induced silencing complex (RISC). As a component of this complex, the mature miRNA regulates the expression oftarget genes by specifically binding to these complementary sequences in the 3'UTR or coding regions of target mRNA, leading to the mRNA degradation or translational repression. Alternatively, the mature miRNA induce the translational activation by specifically binding to the 5'UTR of target mRNA. In addition, miRNAs can be secreted through the exosomal pathway and regulate the gene expression of recipient cells.

During the biogenesis of microRNA, microRNAs are firstly transcribed into pri-microRNAs by RNA polymerase II^18^, then pri-microRNAs are cleaved into pre-microRNAs of 70-80 nucleotides stem-loop by ribonuclease III-Drosha and double strand RNA binding protein-Pasha in nucleus^19^. Next, pre-microRNAs were transported from nucleus to cytoplasm by GTP-dependent exportin5, where pre-microRNAs were cleaved into microRNAs of 8-25 nucleotides mismatch-containing double-stranded RNA by ribonuclease III-Dicer. Double-stranded microRNAs were dissociated^20^-^22^ and integrated into RNA-induced silencing complex (RISC), which forms the asymmetric RISC assembly to bind and regulate the target mRNA^23^,^24^ via the specific binding to the 3'UTR of target mRNAs (Figure 1).

In human genome, 30 percent of genes are regulated by the expression of microRNAs. It's shown that one microRNA can regulate multiple target mRNAs and one mRNA can be regulated coordinately by multiple microRNAs^25^. Future studies on microRNAs may reveal the complexity of *eukaryotic* genomes and the regulation mechanism of gene expression, and provide basis for disease diagnosis, treatment and prognosis.

## miR-30 family

**Figure 2:**
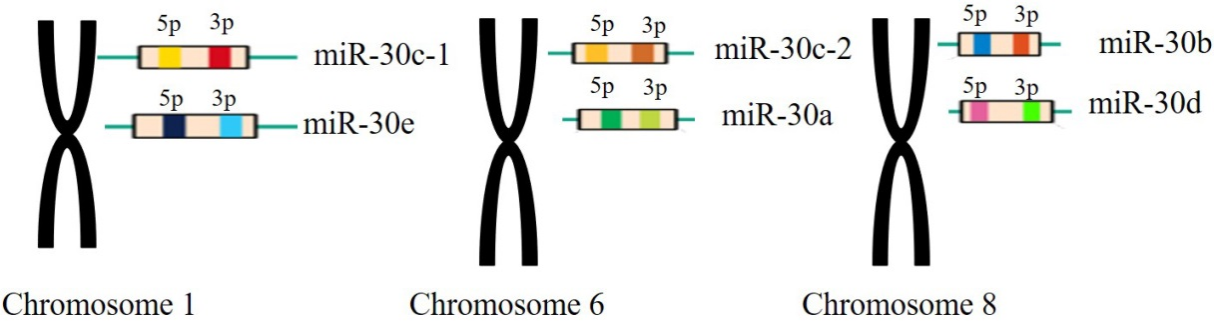
** The miR-30 family members and their genomic locations.** Note: miR-30c-1 and miR-30e are located in the chromosomes 1 withmiR-30c-2 and miR-30a located in the chromosomes 6 and miR-30b, miR-30d located in the chromosomes 8, respectively.

The miR-30 family is an important and complex family that plays vital roles in the function of miRNAs in mammalian and human beings. The miR-30 family contains 5 members and 6 different mature miRNA (miR-30a, -30b, -30c-1, -30c-2, -30d, -30e), which are encoded by 6genes located on human chromosome 1, 6, and 8, respectively^26^(Figure 2).

These miRNAs have the same seed sequence located near the 5' end with different compensating sequences located near the 3' end, which benefit the regulation process of different genes and pathways and sometimes lead to completely opposite behaviors. Consistently, a variety of physiological and pathological conditions* in vivo* are associated with differential expression of miR-30 family members via altering targeted gene expression^27^.In human beings, miR-30c (has-miRNA-30c or miRNA-30c) is encoded by genome 1 and 6 with miR-30c-1, belonging to the same group with miR-30e, encoded by genome 1, and miR-30c-2 encoded by genome 6, respectively. In 2002, Lasgos-Quintana* et al* revealed that miR-30c or mmu-miR-30 was expressed in mouse heart and brain^28^. In 2004, miR-30c was found in TPA-untreated human myeloleukimia cells, demonstrating the existence of mature miR-30c in human cells for the first time^29^. Furthermore, Iwai *et al* revealed that human mature miR-30c was derived from pre-miR-30c-2^30^. In addition, they also found that most mutated pre-miRNA were lost during the process of miRNA maturation, and only mature miR-30c-2 contains theses mutated sequences from pre-miR-30c-2. The heterogeneity of miR-30c may alter target specificity, leading to the altered biological effects.

**Figure 3:**
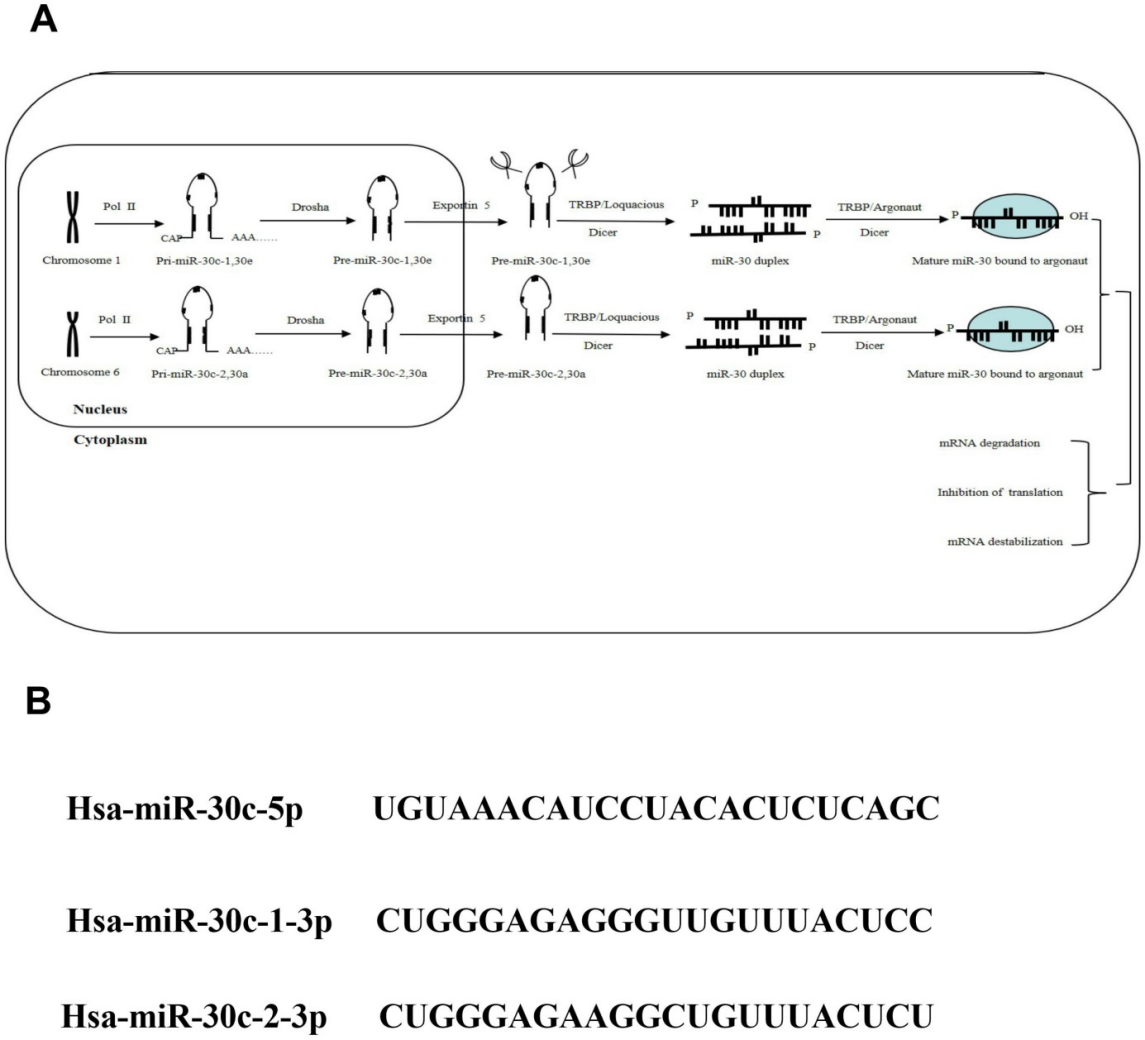
** The biogenesis of miR-30c family.** Note: (A) As important members of miRNA family, the miR-30c family, locatedon human chromosome 1 and 6, contains 2 mature miRNA molecules (miR-30c-1and miR-30c-2). (B) These mature miRNAs share a common seed sequence located near the 5' end with different compensatory sequences located near the 3' end. These different compensatory sequences allow miR-30c family members to target different genes and pathways, thus performing corresponding biological functions.

Recently, overwhelming evidences have suggested that the aberrant expression of miR-30 family is related with multiple cancers progression. As a member of miR-30 family, miR-30c is generally recognized as a multifunctional regulator of cell proliferation, differentiation, metabolism and apoptosis process, which is related with cancer metastasis and chemo-resistance *in vivo*. In this review, the associations between miR-30c dysregulation and cancer progression were investigate to speculate the benefits of miR-30c over-expression in the treatment of cancer (Figure 3).

## miR-30c and cancer progression

### The function of miR-30c in cancer progression

It has been found that miR-30c was low expressed in breast cancer^31^, prostate cancer^32^, colon cancer^33^, medulloblastoma^34^, as well as lung cancer^35^, gastric cancer[36]and multiple myeloma 37, compared with these corresponding adjacent tissues. Increasing research shows miR-30c can be regulated or mutated by oncogenic transcription factors and cancer-associated genes. In 2008, Chang found the repression of multiple miRNA expression in *Myc*-induced B lymphocytes with miRNA chip and RNA blotting. The results showed that after *Myc* induction, low expression of miRNA-30d/miRNA-30b and miRNA-30e/miRNA-30c-1 were detected without the presence of miRNA-30a/miRNA-30c-2. Furthermore, the *Myc* repression byshRNA (short hairpin RNA) tranfection resulted in significant over-expression of multiple miRNAs, including miRNA-30c. These above results demonstrated that expressions of multiple miRNAs, inclding miRNA-30c, were associated with the suppression of *Myc*-induced carcinogenesis^17^. Furthermore, the florescent labeling analysis results of breast cancer progression revealed that miR-30c binds 3'UTP of *KRAS* transcripts and silences the expression of *KRAS* mRNA and protein, which further inhibit the metastasis of breast cancer cells^38^.

In 2012, Zhou found that miRNA-30c repressed the metabolism related gene-*metabolism associated gene 1* (*MTA1*) in Ishikawa (estrogen receptor positive) and HEC-1-B (estrogen receptor negative) endometrial cancer cell lines, and played an inhibitory role in endometrial cancer progression^39^. They further found that over-expression of miR-30c in Ishikawa and HEC-1-B cell lines inhibited the cell proliferation, migration and invasion process. In 2014, Kong provided the direct evidence of miR-30c specific binding to the 3' UTP of *MTA-1*(*Metastasis Associated 1*), which further demonstrated that miR-30c was negatively related with endometrial cancer progression through *MTA-1* inhibition 40.

In 2013, it was reported that the expression levels of miR-30c were significantly increased in various cell lines, especially prostate and breast cancer cell lines, after sulphuretin treatment with decreased expression levels of cyclin D1 and cyclin D2. And the results also proved that miR-30c could specifically bind 3'UTR of cyclin D2^41^.Another study showed that the expression of *FHIT* suppresses the epithelial-mesenchymal transition (EMT) and metastasis of lung cancer through the modulation of miR-30c. The results further showed that miR-30c functions as a negative regulator of EMT and metastasis through directly targeting these mesenchymal markers, including *vimentin*, *fibronectin,* and these metastasis-related genes, including *MTDH* and *HMGA2*, implying that miR-30c contributes to the regulation of EMT and cancer metastasis^42^. During the progression of glioblastoma and lung cancer, miR-30c has been proved to be closely related with tumor necrosis factor (TNF)-related apoptosis-inducing ligand (TRAIL), which caused the apoptosis of cancer cells without killing the normal cells* in vivo.* The results of Quintavalle C's investigation showed that the expression levels of miRNAs, especially miR-30 b/c and miR-21, were significantly increased in the TRAIL-resistant glioma cells. In addition, the related regulation mechanism indicated that the specific bindings between miR-30 b/c ormiR-21 and 3' UTR of *caspase-3*or*TAp63* mRNAs regulated the expressions of downstream protein to produce resistance to TRAIL^43^.In Wu W's research, they found that the migration and invasion abilities of SMMC-7721 and HepG2 liver cancer cells were negatively related with the expression levels of miR-30c through the specific binding between miR-30c and the 3'UTR of *IER2*, resulting in the decreasedIER2 protein expression level, which further affects the cell motilities^44^. In Gong's study, the miRNA expression profiles of natural killer (NK) cells conjugated with either anti-CD226 antibody (LeoA1) or control antibody were analyzed, and the results of miRNA array showed that 6miRNAs were significantly decreased after LeoA1 treatments, including miR-30c and miR-30c-1. Meanwhile, the cytotoxicity of NK cells was enhanced through the inhibition of HMBOX1 via specific binding between miR-30c-1 and 3' UTR of HMBOX1, which leads to the increased expression levels of TNF-α(transmembrane TNF-α, mTNF-α) and repressed liver cancer progression^45^. Jia showed that the ovarian cell proliferation process induced by growth factors mediates a neutralizing response via significantly increased expression levels of miR-30c-2 which resulting in the decreased BCL9 (B-Cell CLL/ Lymphoma 9) expression levels and cell proliferation abilities during ovarian cancer progression^46^. In multiple myeloma cells, the proliferation, apoptosis, invasion, drug resistance and cancer stem cell formation abilities of cancer cells were significantly related with the specific bindings between miR-30c which maintains low expression level and the3'UTR of BCL9, and the following regulation of these downstream genes of Wnt/β-catenin/BCL9 pathways 47. See Table 1.

### The mutation of miR-30c and cancer progression

Some studies showed that the polymorphism mutations ofmiR-30c played an oncogenic role during cancer progression. For instance, Fang suggested that SNPs rs71428439 (miR-149), rs2910164 (miR-146a), rs928508 (mir-30c-1) and rs629367 (let-7a-2) were associated with the lung cancer prevalence, polymorphisms of rs11614913 (miR-196a-2) and rs9280508 (miR-30c-1) significantly influenced the patients' response to platinum-based chemotherapy, which may serve as potential clinical biomarkers to predict lung cancer risk and platinum-based chemotherapy response^55^.However, Yin group found that miR-196a2 rs11614913, miR-30c-1 rs928508, miR-608 rs4919510 and miR-27a rs895819 polymorphisms were not significantly associated with lung cancer risks in any models. The similar results were also found in lung adenocarcinoma patients^56^.Hu demonstrated that the single nucleotide polymorphism (SNP) at rs928508(A/G) of miR-30c flanking region in lung cancer tissues regulated the transformation processes from pri-miR-30c to pre-miR-30c and mature miR-30c, but didn't affect the transcription process of pri-miR-30c, suggesting SNP may alter the process of miRNA maturation, expression and their target-binding abilities, which further leads to lung cancer progression^57^.Another clinical research investigated the associations between SNP of miR-30c in gastric cancer tissues and the risk of cancer recurrence, and the results showed that the expression level of miR-30c was significantly higher in the gastric cancer tissues with the genotype of rs928508 AA than those with GG or AG/GG, and the pre-miR-30c AA genotype was positively associated with lymph node (LN) metastasis of gastric cancer patients^58^.

## The cellular, molecular and drug-resistance mechanisms of MicroRNA-30c in cancer progression and the potential applications of MicroRNA-30c in clinics

### miR-30c as a potential biomarker for cancer patients

In 2010, the miRNA screening results of 246 tamoxifen-treated breast cancer patients with positive estrogen expressions showed that only the expression levels of miRNA-30c, but not miRNA-30a-3p or miRNA-182, could be used as an independent prognostic biomarker for these breast cancer patients after tamoxifen treatment. The results also showed that these breast cancer patients with higher expression levels of miRNA-30c had better prognosis outcomes after tamoxifen treatment, while those patients with low expression levels of miRNA-30c suffered worse prognosis outcomes. Furthermore, they also found that the expression levels of miRNA-30c may be associated with HER and RACI pathways^59^. Gu analyzed the miRNA expression patterns between 41 NSCLC and 5 normal lung tissues with real-time RT-PCR, and the results showed that after the treatments of tyrosine kinase inhibitors (TKIs), as the first-line drug for NSCLC treatments, the expressions of both miR-30b and miR-30c were significantly down-regulated in lung cancer tissues. Further investigations demonstrated that patients with over-expression levels ofmiR-30b or miR-30c had better prognosis outcomes, suggesting miR-30b or miR-30c may be applied as potential prognostic biomarker for the TKIs treatment of NSCLC patients^53^.

### The cellular, molecular and drug-resistance mechanisms of MiR-30c in cancer progression

In 2013, Ling showed that the over-expression of miR-30c repressed the cell proliferation, invasion and metastasis abilities of prostate cancer via blocking the KRAS-MAPK pathway, meanwhile, the down-regulated expression level of miR-30c predicts the early biochemical recurrence and worse prognosis of prostate cancer patients^60^. In Heinzelmann's research, 53 primary clear cell renal cell carcinoma tissues (ccRCC), 35 distant metastatic tissues from lung, bone, brain, and abdomen, as well as 17 normal kidney tissues were analyzed, and the results confirmed the significantly positive correlation between miR-30c expression and ccRCC metastasis, indicating that miR-30c could be used as a promising prognostic marker for early prediction of ccRCC metastasis and personalized cancer therapy^61^. Katzerke demonstrated that the transcription factor-*CCAAT enhancer binding protein alpha* (*C/EBPalpha*) up-regulates the expression level of miR-30c in acute myeloid leukemia (AML) with*NOTCH1*as the direct target of miR-30c, indicating that miR-30c may be a novel biomarker and therapeutic targets of AML^62^.

**Figure 4:**
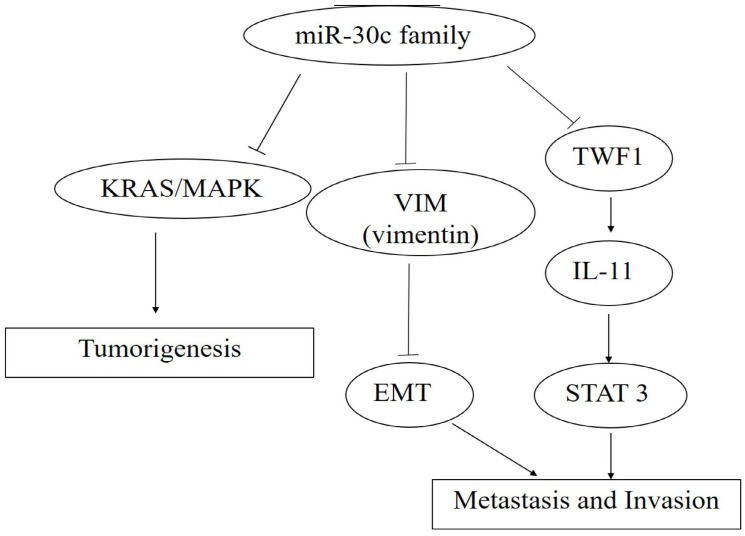
** The roles of miR-30c family members in carcinogenesis, metastasis and invasion.** Note: miR-30c promotes cancer progression through the activation of KRAS/MAPK signal pathway, and miR-30c family members act as caner suppressors by targeting *TWF1* (*Twinfilin actin binding protein 1*)-IL-11-STAT3 pathway. Moreover, cancer metastasis and invasion were inhibited by the decreased EMT process caused by the down-expression leves of*MTDH*(*Metadherin*) and *VIM (Vimentin)* via the specific bindings of miR-30c, respectively.

**Figure 5:**
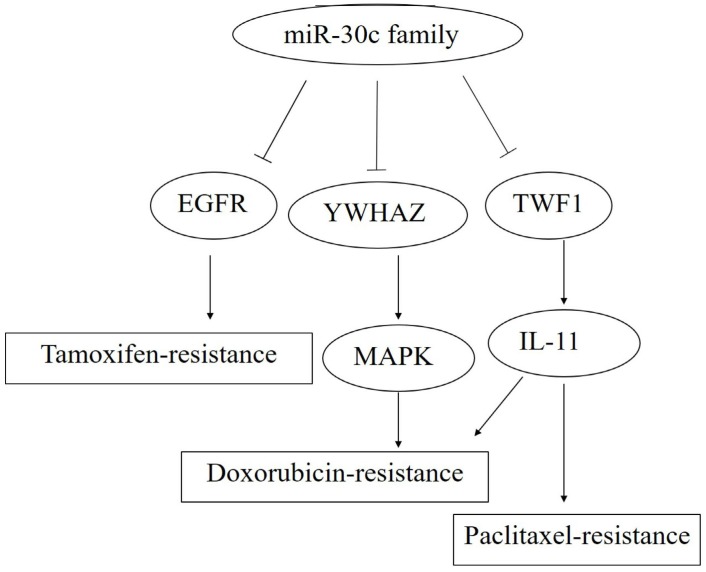
** The roles of miR-30c family members in drug resistance.** Note: miR-30c promotes the paclitaxel- and doxorubicin-resistance by blocking the TWF1-IL-11 pathway.Meanwhile,*YWHAZ* (*Tyrosine 3-Monooxygenase/Tryptophan 5-Monooxygenase Activation Protein Zeta*) was down-regulated by the specific binding with miR-30c, followed bythe activation of MAPK pathway andthe enhanced doxorubicin-resistance. In addition, miR-30c facilitates the tamoxifen resistance through the reduction of EGFR expression.

**Figure 6:**
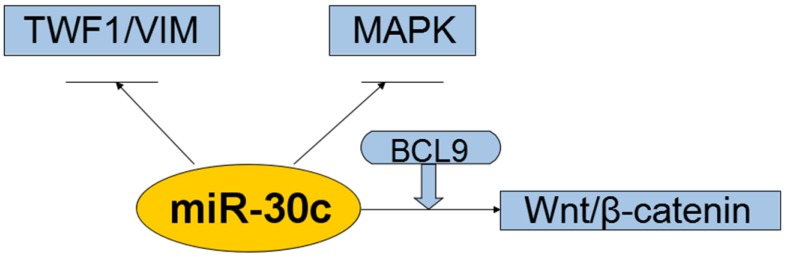
** Interaction of the miR-30c with cellular pathways.miR-30c was down-regulated and exerts tumor-suppressing effects in cancers.** All these suggest that miR-30c may be evolved to protect cells from carcinogenesis in most cases. So far it suggests that related pathways of miR-30c regulation cancers were kRAS/MAPK pathways, Wnt/b-catenin/BCL9 pathways, VIM/TWF1/IL-11 /pSTAT3pathways. Abbreviations: TWF1/VIM: cytoskeleton network genes encoding twinfilin1 and vimentin; MAPK: mitogen-activated protein kinase; BCL9: B-cell CLL/lymphoma 9; miR-30c: microRNA 30c; Wnt: a group of secreted signaling molecules that mediate a variety of cellular processes.

So far, miR-30c has been reported to be targeted and regulated by the transcription factors including *Myc*, *HMBOX1* and C/*EBP alpha* with the target of miR-30cincluding*MTA1*, *KRAS*, BCL-9,*Notch*and cancer cell invasion related genes including *TWF1*, *VIM* and *CCND2*(Figure 4, 5,6).

In Song's research, miR-30c could be use as the potential biomarkers for the diagnosis and prognosis of prostate cancer patients and the detection of miRNAs including miR-30c is an effective way to predict patient's prognosis and evaluate the therapeutic efficacies 63.Liu found that miR-30c impedes glioblastoma cell proliferation and migration by targeting *SOX9*.In their study, they found that the expression levels of miR-30c were significantly down-regulated in glioblastoma tissues and cancer cell lines. This research also found that after miR-30c over-expression, the majority of glioblastoma cells were arrested in G_0_ phase and miR-30c over-expression suppressed the migration and invasion abilities of glioblastoma cells^64^. Ma reported miR-30c functions as a tumor suppressor via targeting *SNAI1* in esophageal squamous cell carcinoma (ESCC). Their results showed that the expression level of miR-30c was significantly down-regulated in ESCC tissues and esophageal cancer cell lines. Due to the significant corrections between low expression level of miR-30c and worse prognosis outcomes of ESCC patients, miR-30c could be used as a promising biomarker and therapeutic target for ESCC in the future^65^.miR-30c has complex roles during the regulation of physiologic and pathologic conditions. Current studies mostly focus on the function of one single miRNA in one specific cancer progression; however, the roles of miRNA vary according to cancer tissue types. Considering multiple targets of one single miRNA and multiple miRNAs targets of the same gene or mRNA, the future researches on miR-30c should use multiple cancer types with large scales to study the related network or complex pathways in order to understand the complex expression and regulation of miRNA-30c in cancer progression.

### The potential applications of miRNA-30c in clinics

miR-30c can suggest clinical prognosis and novel tools for the therapy of various tumors. Busacca group showed the expression of miR-17-5p, miR-21, miR-29a, miR-30c, miR-30e-5p, miR-106a, and miR-143 was significantly associated with the histopathological subtypes. Notably, the reduced expression of two miRNAs (miR-17-5p and miR-30c) correlated with better survival of patients with sarcomatoid subtype. They pointed at miRNAs as potential diagnostic and prognostic markers of mesothelioma, and suggests novel tools for the therapy of this malignancy 66.Quintavalle showed that high expression levels of miR-21 and -30b/c are needed to maintain the TRAIL-resistant phenotype, thus making these miRs as promising therapeutic targets for TRAIL resistance in glioma 67. Egeland suggested that seven microRNAs (miR-10a, miR-26, miR-30c, miR-126a, miR-210, miR-342 and miR-519a) played a role in tamoxifen resistance. Ingenuity Pathway Analysis (IPA) reveals that these seven microRNAs interact more readily with estrogen receptor (ER)-independent pathways than ER-related signaling pathways. Some of these pathways were targetable (e.g., PIK3CA), suggesting that microRNAs as biomarkers of endocrine resistance may have clinical value. Validation of the role of these candidate microRNAs in large prospective studies was warranted 68.

## Conclusions

miR-30c maintains low expression levels in a variety of human cancer tissues and plays an anti-cancer role in cancer progression. However, more research is needed to confirm the functions of miR-30c in different tissues, organs and cancer progressions. Since these endogenous miRs regulate multiple pathways, it is necessary to investigate the various pathways and carefully over-interpret the beneficial effects of regulating one pathway. With the maturity of molecular biology and genetic engineering technology, the prognosis role of miR-30c will benefit the prevention, diagnosis and treatment of various diseases including cancer in the near future.

There are still many key issues that need to be further explained in the future, including (a) the specific roles and regulation mechanisms of different miR-30c family members in different cancer progressions, (b) the differences among miR-30c family members to perform subsequent targeted treatments, (c) the specific role of miR-30c family members in immune organ development and disease-related immune responses.

## Figures and Tables

**Table 1:** Role of miR-30c in human cancers

Cancer	Expression	Target	Function& Biomarker	Reference
**Myc-induced B cell lymphoma**		Unknown	Tumor-suppressing	17
**Breast**	Decreased	KRAS	Inhibits metastasis	48
		VIM/TWF1	Inhibits invasion;a potential sensitive chemotherapy biomarker	38
		HER/RACI	A potential tamoxifengood prognosis biomarker	49
**Colon**	Decreased	ADAM19	Inhibit cells proliferation , metastasis and invasion	33
**Bladder**	Decreased	Unknown	Tumor-suppressing	50
**Medulloblastoma**	Decreased	Unknown	Tumor-suppressing	34
**Lung**	Decreased	Rab18	Inhibited proliferation	51
		Vimentin ,Fibronectin, and MTDH , HMGA2	Inhibited metastasis	52
		Tyrosine kinases	A potential tyrosine kinase inhibitor sensitivity biomarker	53
**Endometrium**	Decreased	MTA1	Tumor-suppressing, inhibited clles growth, migration and invasion,	39,40
**Prostate**	Decreased	CCND2	Tumor-suppressing and a potential Sulfuretin-sensitive biomarker	41
		Possibly kRAS/MAPK	Inhibited cell growth, invasion and metastasis.A potential prognosis biomarker	
**Liver**	Decreased	HMBOX1	IFN-alfal and NK cell cytotoxicity increase	45
		IER2	Inhibited cell motility	44
**Ovarian**	Decreased	BCL9	Inhibited cell proliferation	46
**large cell lymphoma (C-ALCL)**	Increased	Unknown	A different contribution to the pathogenesis of these lymphomas.	54
**multiple myeloma**	Decreased	BCL9	Influence tumor cell proliferation, apoptosis, transfer, drug resistance and tumor stem cell formation	37
